# Structural basis for the interaction of protein S1 with the *Escherichia coli* ribosome

**DOI:** 10.1093/nar/gku1314

**Published:** 2014-12-15

**Authors:** Konstantin Byrgazov, Irina Grishkovskaya, Stefan Arenz, Nicolas Coudevylle, Hannes Temmel, Daniel N. Wilson, Kristina Djinovic-Carugo, Isabella Moll

**Affiliations:** 1 Department of Microbiology, Immunobiology and Genetics, Max F. Perutz Laboratories, Centre for Molecular Biology, University of Vienna, Dr. Bohrgasse 9/4, 1030 Vienna, Austria; 2 Department of Structural and Computational Biology, Max F. Perutz Laboratories, Centre for Molecular Biology, University of Vienna, Campus Vienna Biocenter 5, A-1030 Vienna, Austria; 3 Gene Center, Department of Biochemistry and Center for integrated Protein Science Munich (CiPSM), Ludwig-Maximilians-Universität München, Feodor-Lynen-Strasse 25, 81377 Munich, Germany; 4 Department of Biochemistry, Faculty of Chemistry and Chemical Technology, University of Ljubljana, Aškerčeva 5, 1000 Ljubljana, Slovenia

## Abstract

In Gram-negative bacteria, the multi-domain protein S1 is essential for translation initiation, as it recruits the mRNA and facilitates its localization in the decoding centre. In sharp contrast to its functional importance, S1 is still lacking from the high-resolution structures available for *Escherichia coli* and *Thermus thermophilus* ribosomes and thus the molecular mechanism governing the S1–ribosome interaction has still remained elusive. Here, we present the structure of the N-terminal S1 domain D1 when bound to the ribosome at atomic resolution by using a combination of NMR, X-ray crystallography and cryo-electron microscopy. Together with biochemical assays, the structure reveals that S1 is anchored to the ribosome primarily *via* a stabilizing π-stacking interaction within the short but conserved N-terminal segment that is flexibly connected to domain D1. This interaction is further stabilized by salt bridges involving the zinc binding pocket of protein S2. Overall, this work provides one hitherto enigmatic piece in the ′ribosome puzzle′, namely the detailed molecular insight into the topology of the S1–ribosome interface. Moreover, our data suggest novel mechanisms that have the potential to modulate protein synthesis in response to environmental cues by changing the affinity of S1 for the ribosome.

## INTRODUCTION

During the last decades the bacterial ribosome was at the centre of numerous research efforts that made great strides in elucidating the structure of the translational machinery and the process of protein synthesis at the molecular level. In sharp contrast, protein S1, which is essential for translation initiation in Gram-negative bacteria (1), is still lacking from the high-resolution structures available for *Escherichia coli* and *Thermus thermophilus* ribosomes (2). The protein associates late during assembly of the 30S ribosomal subunit (3) and interacts with a pyrimidine-rich region in the 5′ untranslated region (5′UTR) of mRNAs (4). Here, S1 unwinds RNA structures by binding to single-stranded RNA during thermal breathing (5). Thus, the protein shows RNA chaperone activity (6) and is essential for the binding and the accommodation of structured mRNAs into the decoding channel (7). Notably, S1 is dispensable for translation of leaderless mRNAs (lmRNAs) that lack a 5′UTR and hence harbour a 5′-terminal AUG start codon (8,9).

**Figure 1.**
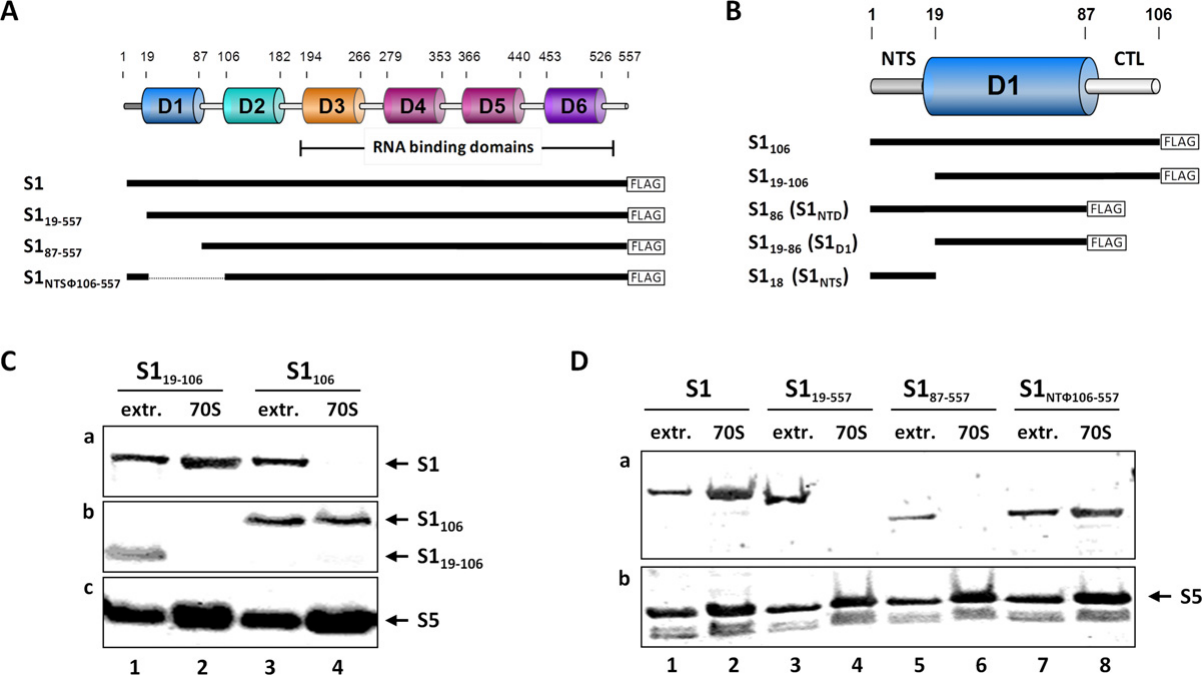
The N-terminal segment is essential for protein S1 to interact with the ribosome *in vivo*. (**A**) Schematic of the domain organization of protein S1 and the C-terminally FLAG-tagged S1 variants used in the study. (**B**) The N-terminal domain D1 of protein S1 including the flexible N-terminal segment (NTS) and the C-terminal linker (CTL) is enlarged, and its variants used in the study are depicted below. (**C**) Equimolar amounts of S30 extracts (lanes 1 and 3) and 70S ribosomes (lanes 2 and 4) purified from *E. coli* strain JE28 synthesizing protein S1_19_–_106_ (lanes 1 and 2) or protein S1_106_ (lanes 3 and 4) were analysed for the presence of native S1 (panel a) and proteins S1_106_ and S1_19_–_106_ (panel b) by western blotting using antibodies directed against S1_106_ (18). Western blotting of protein S5 served as loading control (panel c). (**D**) Equal amounts of S30 extracts (extr.; lanes 1, 3, 5 and 7) and ribosomes (70S; lanes 2, 4, 6 and 8) purified from *E. coli* strain JE28 upon synthesis of FLAG-tagged proteins S1 (lanes 1 and 2), S1_19_–_557_ (lanes 3 and 4), S1_87_–_557_ (lanes 5 and 6) or S1_NTФ106–557_ (lanes 7 and 8) were analysed for the presence of the respective proteins by western blotting employing anti-FLAG antibodies (panel a). Protein S5 served as loading control (panel b).

Structurally, S1 is composed of six contiguous domains (D1–D6; Figure 1A), which are connected *via* linkers providing the flexibility that is likely to play a role in recruitment of mRNA transcripts to the ribosome (10). The structural organization of the single C-terminal domains (D3–D6; Figure 1A), which interact with ssRNA (4,11), was modelled for D3, D4 and D5 (12) and, later on, solved at atomic resolution for D4 and D6 (13). Each of these domains displays an oligosaccharide–oligonucleotide binding (OB)-fold, consisting of two three-stranded antiparallel β-sheets, where strand 1 is shared by both sheets, with an α-helix that packs against the bottom of the barrel, typically oriented lengthwise along the long axis of the β-barrel (14). Immune-electron microscopic studies revealed that domains D3–D6 extend from the platform side of the 30S subunit where the 5′-end of the mRNA would be located (15). In contrast, the two N-terminal domains (D1, D2; Figure 1A) have no detectable RNA-binding activity but rather provide the boundary to the ribosome (11,16). In contrast to studies that suggest a potential interaction of S1 with the 16S rRNA ((17) and references therein), several lines of evidence indicate that the N-terminal region of S1 comprising 106 amino acids (S1_106_) is sufficient to ensure its assembly to the 30S ribosomal subunit (10,18) by means of protein–protein interactions *via* protein S2 (9,19).

Besides its pivotal role in protein synthesis, S1 acts as a host factor component of the replicase holoenzyme of the bacteriophage Qβ (20). Interestingly, this function can be performed by the N-terminal part of the protein comprising domains D1 and D2 (21). During the preparation of this manuscript the structure of the Qβ replicase comprising the β-subunit, EF-Tu, EF-Ts and the N-terminal half of S1 was published revealing that domains D1 and D2 function to anchor S1 on the β-subunit (22). However, the structure of S1 when assembled to the ribosome is unknown. Due to the intrinsic flexibility of the protein, ribosomes were intentionally depleted for S1 to facilitate the crystallization process for structural analyses (2). Thus, the molecular mechanism governing the S1-ribosome interaction has still remained elusive. Nevertheless, the *E. coli* S1 protein was tentatively localized on the ribosome based on difference electron density maps between a cryo-electron microscopy (EM) structure of the *E. coli* 70S ribosome containing S1 and a map based on the crystal structure of the *T. thermophilus* 30S that lacked S1 (23). The results suggested that S1 binds within the cleft at the base of the small subunit head and platform; however, the limited resolution prevented any molecular interpretation.

Here, we present the first crystal structure of the N-terminal domain of protein S1 (comprising 86 amino acid residues; hereafter referred to as S1_NTD_; Figure 1B) in complex with protein S2 at 2.4–3 Å resolution, showing detailed insights into the molecular basis of the S1–ribosome interaction. In addition, we have visualized S1 bound to the ribosome using cryo-EM underpinning the S1–S2 interaction observed in the crystal structure. Together with functional analyses, we demonstrate that a short, but highly conserved, N-terminal segment is the primary ribosome anchoring point for S1. This interaction is further stabilized by salt bridges between the globular fold of S1_NTD_ and S2. Notably, the structure shows that the anchoring helix is connected by a flexible hinge region with domain D1, which mechanistically supports the dynamic movement of S1 when bound to the 30S subunit. Moreover, our functional studies suggest potential mechanisms, which might fine tune the affinity of S1 for the ribosome in response to environmental cues.

## MATERIALS AND METHODS

### Bacterial strains and plasmids

*Escherichia coli* strains, plasmids and oligonucleotides used in this study are listed in the Supplementary Tables S1 and S2. Unless otherwise indicated, bacterial cultures were grown in Luria–Bertani (LB) medium supplemented with ampicillin (100 μg/ml). When appropriate, kanamycin (20 μg/ml) was added. Growth was monitored by measuring the optical density at 600 nm (OD_600_).

### Construction of plasmids

The sequence encoding the HA-tagged version of protein S2 was amplified by polymerase chain reaction (PCR) using primers P1 and P2 employing genomic DNA of *E. coli* strain MG1655 as a template. The respective products were cleaved with NarI and XhoI and cloned under control of the Trc promoter in the corresponding sites of plasmid pProEX–Htb (Life Technologies) resulting in plasmid pProEX–S2–HA. To generate plasmid pProEX–S2–S1_NTD_ for expression of the chimeric protein S2–S1_NTD_, the sequence encoding protein S1_86_ was amplified with primers P3 and P4. The PCR product was cleaved with HindIII and ligated between the two HindIII-sites of the plasmid pProEX–S2–HA. Plasmid pPro-S1F (18) was used as a template to construct plasmid pPro-S1_NTSΦ106–557_F. The coding sequence for the S1 domain D1 was removed employing the Phusion site-directed mutagenesis kit (NEB) using 5′-monophosphorylated primers P5 and P6. Plasmids pPro-S1F and pPro-S1_106_F (18) were used as templates to construct plasmids pPro-S1_19–557_F and pPro-S1_19–106_F. The coding sequence for the N-terminal 18 amino acids was removed employing the Phusion site-directed mutagenesis kit (NEB) using 5′-monophosphorylated primers P7 and P8. Plasmids pPro-S1_106_F and pPro-S1_19–106_F were used as templates to construct plasmids pPro-S1_86_F and pPro-S1_19–86_F. The coding sequence for the C-terminal 18 amino acids was removed employing the Phusion site-directed mutagenesis kit (NEB) using 5′-monophosphorylated primers P9 and P10. The plasmid pPro-S1_86_F was used as a template to construct plasmids pPro-S1_86_F encoding variants of S1_NTD_ harbouring the F5A, F9A, D39K and K43E mutations. The respective mutations were introduced employing the Phusion site-directed mutagenesis kit (NEB) using the 5′-monophosphorylated primers P15/P17, P16/P17, P18/P20 and P19/P20, respectively. The sequences encoding proteins S1_106_, S1_86_ and S1_19–86_ were amplified by PCR employing pairs of primers P11/P13, P11/P14 and P12/P14 respectively. The respective products were cleaved with NdeI and XhoI and cloned under control of the T7 promoter in the corresponding sites of plasmid pET22b (Novagen) yielding plasmids pET-S1_106_, pET-S1_86_ and pET-S1_19–86_. All constructs were verified by sequencing (Microsynth).

### Overexpression and purification of the chimeric protein S2-S1_NTD_

*Escherichia coli* strain Tuner harbouring plasmid pProEX–S2–S1_NTD_ was grown in LB medium at 37°C. Expression of the *rpsB*–*rpsA_NTD_* fusion gene was induced by addition of 1 mM Isopropyl-D-thiogalactopyranoside (IPTG). The HIS-tagged chimeric protein S2–S1_NTD_ was purified with the TALON cobalt resin (Clontech) and subsequently treated with AcTEV-protease (Life Technologies) and purified *via* a HiLoad 26/60 Superdex 200 column (GE Healthcare) to remove the His-tag using a buffer containing 100 mM HEPES (2-[4-(2-hydroxyethyl)piperazin-1-yl]ethanesulfonic acid)–KOH, (Potassium hydroxide) pH 7.4, 6 mM MgCl_2_ and 200 mM KCl. The fractions containing the chimeric protein were concentrated with an Amicon ultra centrifugal filter unit (MWCO of 30 kDa; Millipore).

### Crystallization, data collection, structure determination and refinement

Crystals of the S2–S1_NTD_ chimeric construct were initially obtained in the crystallization screen JBScreen 7 (Jena Bioscience), using the sitting-drop vapor diffusion technique and a nanodrop-dispensing robot (Phoenix RE; Rigaku Europe, Kent, United Kingdom). Crystallization conditions were optimized to 0.1 M HEPES–KOH, pH 7.4, 3 mM MgCl_2_, 7.5% (w/v) PEG 6000, 3% (w/v) 2-methyl-pentanediol-2,4, 100 mM KCl using the hanging drop vapor diffusion technique at 22°C. The crystals were flash cooled in liquid nitrogen prior to data collection. The data set has been collected at the beamline I04 of the Diamond Light Source at 100 K using a wavelength of 0.98 Å. The data frames were processed using the XDS package (24), and converted to the mtz format with the program AIMLESS (25). In assessing the data quality and establishing the resolution cutoff we relied on criteria based on the correlation coefficient CC1/2 (26). The structure was solved by using the molecular replacement pipeline program BALBES (27), the log file indicated that atomic coordinates of S2 from 30S subunit of *E. coli* (pdb accession code: 2qbf, chain B) and the fragment of hypothetical protein PA5201 from *P. aeruginosa* (pdb accession code: 2oce) were yielded in solution. About 90% of the model was placed using the program AUTOBUILD from Phenix software package (28). The structure was then refined with the REFMAC (29) and Phenix Refine (28) and finally, the rebuilding of structure was done using the program Coot (30). Stereochemistry and structure quality were also checked using the program MolProbity (31). The figures were produced using the Pymol software (32). Coordinates have been deposited in the protein data bank (pdb accession code: 4toi). Data collection and refinement statistics are reported in Table 1.

**Table 1.** Data collection and refinement statistics

Data collection	
Source	I04, Diamond
Wavelength (Å)	0.98
Resolution (Å)	37.79–2.30
	(2.38– 2.30)^a^
Space group	*P*3_1_21
Unit cell (Å, °)	*a* = *b* = 87.28
	*c* = 94.36; *α* = *γ* = 90; *β* = 120
Molecules (a.u.)	1
Unique reflections	18 726 (1670)
Completeness (%)	99.0 (91.3)
*R*_merge_^b^	0.175 (1.106)
*R*_meas_^c^	0.186 (1.238)
*R*_pim_^d^	0.063 (0.543)
Multiplicity	8.1 (4.5)
*I*/sig(*I*)	7.4 (1.5)
CC (1/2)	0.993 (0.548)
*B*_Wilson_ (Å^2^)	22.1

Refinement	
*R*_cryst_^e^/*R*_free_^f^ (%)	16.3/22.8
rmsd bonds (Å)	0.008
rmsd angles (º)	1.09

^a^Values in parentheses are for the highest resolution shell.

^b^$R_{{\rm merge}} = \frac{{\sum\limits_{hkl} {\sum\limits_{i = 1}^N {\left| {I_{i(hkl)} - \bar I_{(hkl)} } \right|} } }}{{\sum\limits_{hkl} {\sum\limits_{i = 1}^N {I_{i(hkl)} } } }}$

^c^$R_{{\rm meas}} = \frac{{\sum\limits_{hkl} {\sqrt {N/(N - 1)} } \sum\limits_{i = 1}^N {\left| {I_{i(hkl)} - \bar I_{(hkl)} } \right|} }}{{\sum\limits_{hkl} {\sum\limits_{i = 1}^N {I_{i(hkl)} } } }}$

^d^$R_{{\rm pim}} = \frac{{\sum\limits_{hkl} {\sqrt {1/(N - 1)} } \sum\limits_{i = 1}^N {\left| {I_{i(hkl)} - \bar I_{(hkl)} } \right|} }}{{\sum\limits_{hkl} {\sum\limits_{i = 1}^N {I_{i(hkl)} } } }}$

Where $\bar I_{(hkl)}$ is the mean intensity of multiple $I_{i(hkl)}$ observations of the symmetry-related reflections, *N* is the redundancy.

^e^$R_{{\rm cryst}} = \frac{{\sum {\left| {|F_{{\rm obs}} | - |F_{{\rm calc}} |} \right|} }}{{\sum {|F_{{\rm obs}} |} }}$

^f^*R*_free_ is the cross-validation *R*_factor_ computed for the test set of reflections (5%) which are omitted in the refinement process.

### Purification of ribosomal subunits

Ribosomal subunits were purified based on the His-tagged proteins L7/L12 employing Ni-NTA-agarose (33). Briefly, *E. coli* strain JE28 was grown in LB medium supplemented with kanamycin (20 μg/ml). At OD_600_ 0.7–0.9 the culture was harvested by centrifugation at 5000g for 20 min at 4°C. The cell pellet was resuspended in lysis buffer (20 mM Tris·HCl, pH 7.4, 10 mM MgCl_2_, 30 mM NH_4_Cl, 100 mM KCl, 10 mM Imidazole, 1 unit/ml RNase-free DNase I (Roche), 0.1 mM PMSF(phenylmethanesulfonylfluoride)). The cells were disrupted by three freeze and thaw cycles and the lysate was cleared by centrifugation at 15 000g for 20 min at 4°C. The extracts were applied to 10 ml of Ni-NTA-agarose (QIAGEN) pre-equilibrated in lysis buffer, and washed by 10 column volumes of washing buffer (20 mM Tris·HCl, pH 7.4, 10 mM MgCl_2_, 30 mM NH_4_Cl, 150 mM KCl, 20 mM Imidazole). Thereafter, the Ni-NTA-agarose was resuspended in 10 column volumes of dissociation buffer (20 mM Tris·HCl, pH 7.4, 1 mM MgCl_2_, 30 mM NH_4_Cl, 150 mM KCl, 20 mM Imidazole) and incubated for 8 h at 4°C. The flow-through fractions that contain the 30S ribosomal subunits were collected and the Mg^2+^ concentration was adjusted to 10 mM. The tetra-His-tagged 50S subunits were eluted by 10 column volumes of elution buffer (20 mM Tris·HCl, pH 7.4, 10 mM MgCl_2_, 30 mM NH_4_Cl, 150 mM KCl, 150 mM Imidazole). The fractions containing ribosomal subunits were dialysed against tight-couple (TICO) buffer (20 mM HEPES–HCl pH 7.6, 6 mM MgCl_2_, 30 mM NH_4_Cl and 4 mM β-mercaptoethanol) and concentrated using Amicon filter devices (MWCO of 100 kDa; Millipore). Protein S1-depleted 30S ribosomes were prepared by affinity chromatography using poly(U)-Sepharose 4B (Pharmacia) (34).

### Co-purification of tetra-His-tagged ribosomes with FLAG-tagged protein S1 variants

*Escherichia coli* strain JE28 cells harbouring plasmids pProEX–HTb, pPro-S1_86_F, pPro-S1_106_F, pPro-S1_19–86_F, pPro-S1_19–106_F, pPro-S1F, pPro-S1_19–557_F, pPro-S1_87–557_F, pPro-S1_NTSΦ106–557_F, pPro-S1_86_F_F5A_, pPro-S1_86_F_F9A_, pPro-S1_86_F_D39K_ and pPro-S1_86_F_K43E_ were grown in LB broth supplemented with 100 μg/ml ampicillin, 20 μg/ml kanamycin and 0.5% (w/v) glucose. The synthesis of FLAG-tagged protein S1 variants was induced by addition of 0.1 mM IPTG at OD_600_ of 0.30–0.35. One hour thereafter the cells were harvested by centrifugation and lysed by three freeze and thaw cycles in lysis buffer containing 20 mM Tris·HCl, pH 7.4, 10 mM MgCl_2_, 30 mM NH_4_Cl, 100 mM KCl, 10 mM Imidazole, 0.1 mM PMSF, 1 unit/ml RNase-free DNase I (Roche). After centrifugation at 30 000g for 30 min at 4°C, the extracts were applied to the Ni-NTA agarose (QIAGEN), washed by 10 column volumes of washing buffer (20 mM Tris·HCl, pH 7.4, 10 mM MgCl_2_, 30 mM NH_4_Cl, 150 mM KCl, 20 mM Imidazole) followed by elution with a buffer containing 20 mM Tris·HCl, pH 7.4, 10 mM MgCl_2_, 30 mM NH_4_Cl, 150 mM KCl, 150 mM Imidazole. The protein composition of the ribosomes was determined by western blot analysis using anti-FLAG (Abcam), anti-S1_106_ and anti-S5 antibodies.

### Purification of ^15^N-labelled proteins S1_106_, S1_86_ and S1_19–86_

*Escherichia coli* strain Tuner (DE3) harbouring plasmids pET-S1_106_, pET-S1_86_ and pET-S1_19–86_ were grown in M9 minimal medium supplemented with ^15^NH_4_Cl (Sigma, 1 g/l) and 100 μg/ml ampicilin. The synthesis of the respective proteins was induced by addition of 1 mM IPTG at OD_600_ of 0.8–0.9. Two hours thereafter the cells were harvested by centrifugation and lysed by three freeze and thaw cycles in lysis buffer containing 50 mM Na_2_HPO_4_, pH 8.0, 300 mM NaCl, 10 mM Imidazole, 0.1% Tween-20, 0.5 mg/ml DNase I (Roche), 20 μg/ml RNase A. After centrifugation at 4°C, 30.000g for 30 min, the S30 extracts were applied to a Ni-NTA agarose, washed by 10 column volumes of washing buffer (50 mM Na_2_HPO_4_, pH 8.0, 500 mM NaCl, 20 mM Imidazole) followed by elution with elution buffer (50 mM Na_2_HPO_4_, pH 8.0, 300 mM NaCl, 250 mM imidazole). The eluted fractions were dialysed against phosphate buffered saline (PBS) buffer and a size exclusion fast protein liquid chromatography (FPLC) was performed on a HiLoad Sephadex 75 16/60 column (GE Healthcare). The purified proteins were concentrated using Amicon filter devices (MWCO of 3 kDa; Millipore).

### Purification of protein S2-HA

*Escherichia coli* strain Tuner harbouring plasmid pProEX–S2-HA was grown in M9 minimal medium supplemented with 100 μg/ml ampicillin. The synthesis of protein S2-HA was induced by addition of 0.1 mM IPTG at OD_600_ of 0.5–0.6. Four hours later, the cells were harvested and lysed by three freeze and thaw cycles in lysis buffer containing 50 mM HEPES–KOH, pH 7.4, 3 mM MgCl_2_, 200 mM KCl, 10 mM imidazole, 0.1% Tween-20, 0.5 mg/ml DNase I (Roche), 20 μg/ml RNase A. After centrifugation at 100 000g for 30 min at 4°C, the supernatant was applied to Ni-NTA agarose, washed by 10 column volumes of washing buffer (50 mM HEPES–KOH, pH 7.4, 3 mM MgCl_2_, 250 mM KCl, 10% (w/v) glycerol, 20 mM imidazole) followed by elution with elution buffer (50 mM HEPES–KOH, pH 7.4, 3 mM MgCl_2_, 200 mM KCl, 200 mM imidazole). The eluted proteins were dialysed against 50 mM HEPES–KOH, pH 7.4, 3 mM MgCl_2_, 150 mM KCl.

### Co-purification analysis

Purified protein S2–HA was incubated with S100 extracts prepared from the *E. coli* strain Tuner over-expressing the different *rpsA* genes as follows. *Escherichia coli* cells carrying the plasmids pProEX–HTb, pPro-S1_19–86_F or plasmids pPro-S1_86_F containing the different point mutations (WT, F5A, F9A, D39K or K43E) were grown in LB-Amp (100 μg/ml). 30 min after addition of 0.1 mM IPTG at OD_600_ of 0.4–0.5 the cells were harvested and lysed by three freeze and thaw cycles in lysis buffer (50 mM HEPES–KOH, pH 7.4, 3 mM MgCl_2_, 200 mM KCl, 0.1% Tween-20, 0.5 mg/ml DNase I (Roche)). After centrifugation at 100 000g for 60 min at 4°C, the amount of S1 protein variants was determined by quantitative western blotting. The extracts were combined with equimolar amounts of protein S2–HA and incubated at 37°C for 30 min. Co-immunoprecipitation was performed employing anti-ECS antibodies (Bethyl) covalently linked to protein A magnetic beads (Life Technologies). After three washing cycles (50 mM HEPES–KOH, pH 7.4, 3 mM MgCl_2_, 100 mM KCl) the proteins were eluted from the beads by boiling in Laemmli buffer, separated on sodium dodecyl sulphate-polyacrylamide gel electrophoresis (SDS-PAGE) and analysed by western blotting employing anti-FLAG (Abcam), anti-HA (Sigma) and anti-S1_106_ antibodies.

### Generation and purification of ErmCL-SRC

The *2XermCL* construct was synthesized (Eurofins, Martinsried, Germany) such that it contained a T7 promoter followed by a strong ribosome binding site (RBS) spaced by seven nucleotides (nts) to the ATG start codon of the first *ermCL* cistron. A linker of 22 nts separated the stop codon of the first *ermCL* cistron and the start codon of the second *ermCL* cistron. The linker also comprised the strong RBS 7 nts upstream of the ATG start codon of the second *ermCL* cistron, enabling initiation of translation independent from the first *ermCL* cistron. Each *ermCL* cistron encoded amino acids 1–19 corresponding to ErmCL leader peptide (GenBank accession number: V01278) present on macrolide resistance plasmid pE194 (35). The complete sequence of *2XermCL* construct is:

5′-*TAATACGACTCACTATAGGG*AGTTTTATA**AGGAGG**AAAAAATatgggcattttta**gta**tttttgtaatcagcacagttcattatcaaccaaacaaaaaataaAGTTTTATA**AGGAGG**AAAAAATatgggcattttta**gta**tttttgtaatcagcacagttcattatcaaccaaacaaaaaataa-3′ (T7 promoter, italics; RBS, bold; ErmCL ORF, small letters with GTA codon in P-site of stalled ribosome shown in bold; Annealing site for complementary DNA oligonucleotide, underlined). *In vitro* translation of the *2xermCL* construct was performed using the Rapid Translation System RTS 100 *E. coli* HY Kit (Roche; Cat. no. 3246817). Translations were carried-out in the presence of 10 μM erythromycin (ERY) for 1 h at 30°C. Control reactions were performed in the absence of erythromycin as well as using a monocistronic *ermCL* construct. Translation reactions were analysed on sucrose density gradients (10–55% sucrose in a buffer A, containing 50 mM HEPES–KOH, pH 7.4, 100 mM KOAc, 25 mM Mg(OAc)_2_, 6 mM β-mercaptoethanol, 10 μM erythromycin and 1× Complete EDTA-free Protease Inhibitor cocktail (Roche)) by centrifugation at 154 693g (SW-40 Ti, Beckman Coulter) for 2.5 h at 4°C. For ErmCL–SRC purification, disome fractions were collected using a Gradient Station (Biocomp) with an Econo UV Monitor (Biorad) and a FC203B Fraction Collector (Gilson). Purified ErmCL–SRC disomes were concentrated by centrifugation through Amicon Ultra-0.5 ml Centrifugal Filters (Millipore) according to the manufacturer's protocol. To obtain monosomes of the ErmCL–SRC, a short DNA oligonucleotide (5′-ttcctccttataaaact-3′, Metabion) was annealed to the linker between the *ermCL* cistrons of the disomes, generating a DNA–RNA hybrid that could be cleaved by RNase H (NEB) treatment at 25°C for 1 h.

### Negative-stain electron microscopy

Ribosomal particles were diluted in buffer A to a final concentration of 0.5 A_260_/ml. One drop of each sample was deposited on a carbon-coated grid. After 30 s, grids were washed with distilled water and then stained with three drops of 2% aqueous uranyl acetate for 15 s. The remaining liquid was removed by touching the grid with filter paper. Micrographs were taken using a Morgagni transmission electron microscope (FEI), 80 kV, wide angle 1 K CCD at direct magnifications of 72 K. The negative stain electron microscopy was used to prescreen the samples and ensure that the concentration was accurate to obtain an optimal density and distribution of ribosomal particles for the cryo-grids.

### Cryo-electron microscopy and single particle reconstruction

Monosomes of the ErmCL–SRC were applied to 2 nm pre-coated Quantifoil R3/3 holey carbon supported grids and vitrified using a Vitrobot Mark IV (FEI Company). Data collection was performed on a Titan Krios transmission electron microscope (TEM) (FEI, Netherlands) under low-dose conditions (∼20 e^−^/Å^2^) at a nominal magnification of 75 000× with a nominal defocus between -1 and -3.5 μm. Images were collected at 200 keV at a magnification of 148 721× at the plane of CCD using a TemCam-F416 CMOS camera (TVIPS GmbH, 4096 × 4096 pixel, 15.6 μm pixel, 1 s/full frame), resulting in an image pixel size of 1.0489 Å (object scale). Data collection was facilitated by the semi-automated software EM-TOOLS (TVIPS GmbH) as described (36). Contrast-transfer functions were determined using the SPIDER TF ED command and recorded images were manually inspected for good areas and power-spectra quality. Data were processed further using the SPIDER software package (37), in combination with an automated workflow as described previously (36). After initial, automated particle selection based on the program SIGNATURE (38) initial alignment was performed with 624 304 particles, using *E. coli* 70S ribosome as a reference structure (39). After removal of noisy particles (76 346 particles; 12%) and non-aligning particles (271 873 particles; 44%), the dataset could be sorted into two main subpopulations: The first subpopulation (153 240 particles; 25%) was defined by the presence of non-stoichiometric densities for tRNAs in the A-, and P-sites. The second, homogeneous subpopulation was defined by the absence of density for the A-tRNA and the presence of stoichiometric density for the P-tRNA (128 846 particles; 21%). This major subpopulation could be refined to an average resolution of 7.9 Å according to the Fourier Shell Correlation (FSC at a cut-off value of 0.5).

### Molecular modeling and map-docking procedures

**Figure 2.**
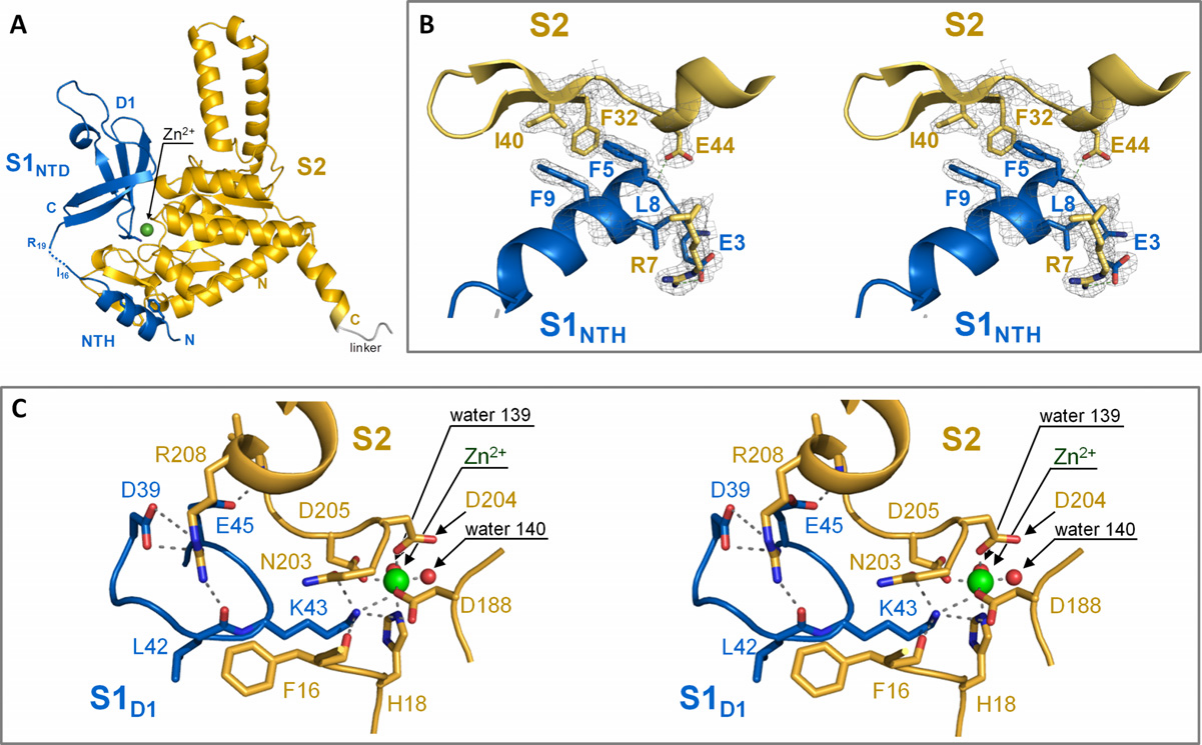
Interaction between S1_NTD_ and protein S2. (**A**) Overview showing the S2–S1_NTD_ complex structure assembled from two protomers, with S1_NTD_ in blue, S2 in yellow. Zn^2+^ is depicted as a green sphere. This colour code is used throughout the figures. (**B**) Stereo view showing the close up of the π-stacking interaction with the aromatic ring of Phe32 of protein S2 with Phe5 and Phe9 of S1_NTH_. (**C**) Stereo view showing the salt bridge interactions between the core domain S1_D1_ and the globular domain of S2 involving the Zn^2+^ binding pocket. The water molecules involved in the coordination of the Zn^2+^ ion are shown as red spheres.

**Figure 3.**
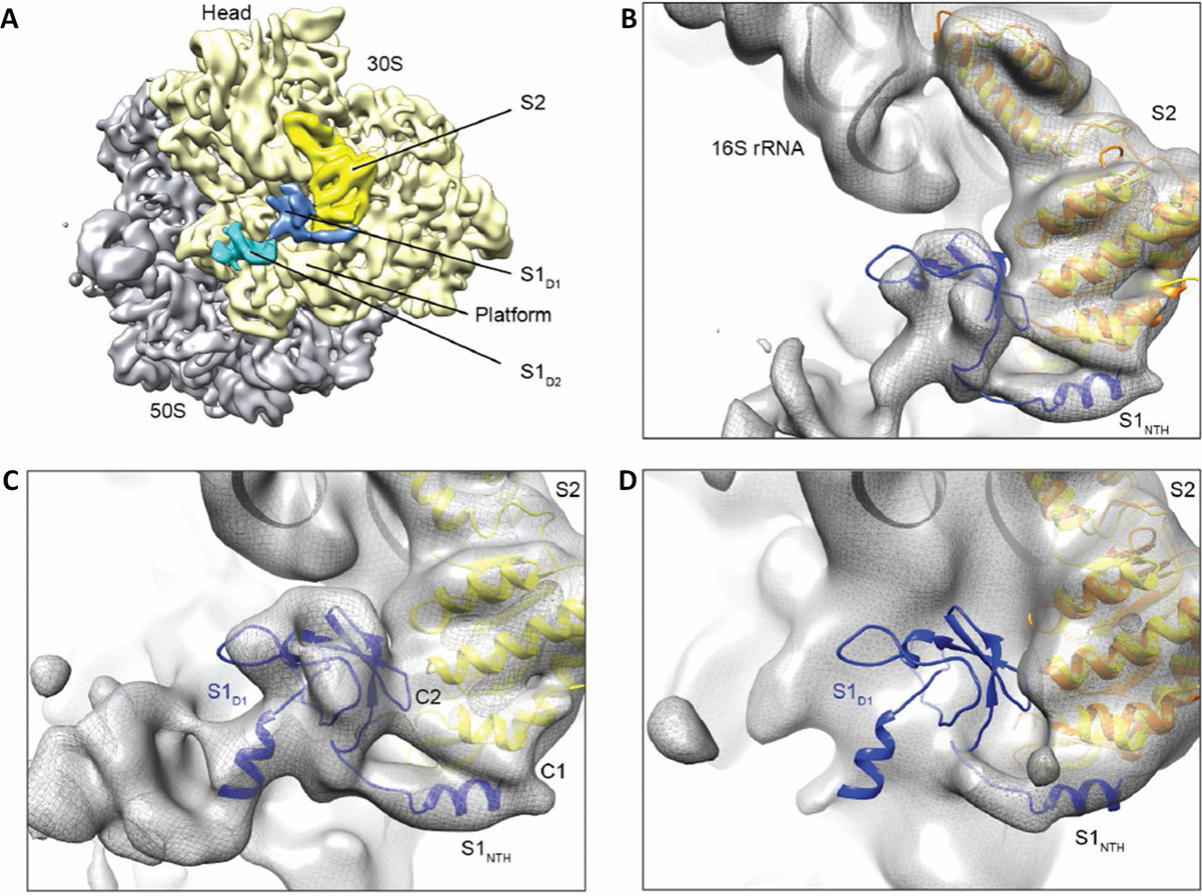
Binding position of S1 on the *E. coli* 70S ribosome. (**A**) Cryo-EM structure of a translating *E. coli* 70S ribosome containing additional density for domain 1 (S1_D1_, blue) and domain 2 (S1_D2_, cyan) of ribosomal protein S1. Density for the large (grey) and small (pale yellow) ribosomal subunit, together with ribosomal protein S2 (bright yellow) is indicated. (**B**) Initial model for the position of S1_NTD_ obtained by aligning S2 (yellow) of the chimeric S2–S1_NTD_ with S2 (orange) from an *E. coli* 30S subunit (pdb accession code: 3ofo (40)) fitted to the cryo-EM map (grey mesh) as a rigid body. (**C**) Refined model for the complete S1_NTD_ based on homology with eIF2α (pdb accession code: 1kl9 (43)) and fitted so as to maintain interactions between S1 and S2 as observed in the chimeric crystal structure, but also constrained by the electron density of the cryo-EM map (grey mesh). (**D**) The position of S1_NTD_ (blue) relative to the *E. coli* 70S ribosome at 11 Å (EMD-1003 (45)) based on aligning S2 (yellow) of the chimeric S2–S1_NTD_ with S2 (orange) from an *E. coli* 30S subunit (pdb accession code: 3ofo (40)) fitted to the cryo-EM map (grey mesh) as a rigid body.

The crystal structure of the 30S subunit (pdb accession code: 3ofo) of the *E. coli* 70S ribosome (40) was fitted as a rigid body into the cryo-EM density map of the ErmCL–SRC using UCSF Chimera (41) (fit in map function). The molecular model for S1_D1_ was based on the crystal structure of the S2–S1_NTD_ complex (Figure 2) and the C-terminus of S1 was extended (amino acids V68-A105) based on a homology model generated by HHPred (42) using the crystal structure of eIF2α as a template (pdb accession code: 1kl9 (43)). Upon alignment of S2 of the S2–S1_NTD_ complex to S2 of *E. coli* 30S subunit (pdb accession code: 3ofo (40)), S1_D1_ fitted nicely into additional density on the ErmCL–ribosome complex (Figure 3C). The final model was adjusted manually using Coot (30) to fit the density of the ErmCL–ribosome map. Crystal structures of 30S subunits from *T. thermophilus* (pdb accession code: 1j5e (44)) and *E. coli* (pdb accession code: 3ofo (40)) were filtered to comparable resolutions using the Molmap function in Chimera. Difference electron density maps were then calculated in SPIDER (37) by subtracting the filtered map for *T. thermophilus* 30S subunit (pdb accession code: 1j5e (44)) or *E. coli* 30S subunit (pdb accession code: 3ofo (40)) from either EMD-1003 (45) or the ErmCL–SRC map (Supplementary Figure S4A–D).

### Figure preparation

Figures showing electron densities and atomic models were generated using UCSF Chimera (41) or Pymol (http://www.pymol.org/) (32).

### *In vitro* binding of FITC-labelled peptide S1_18_ to the 30S(-S1) subunit

The FITC–S1_18_ peptide (FITC–MTESFAQLFEESLKEIE-COOH) was synthesized by Fmoc *N*-(9-fluorenyl)-methoxycarbonyl solid-phase peptide synthesis and N-terminally labelled with fluorescein isothiocyanate (FITC). The average molecular mass of the peptide was determined to be 2254 Da with an Applied Biosystems Voyager System 1105 mass spectrometer. 40 pmol of 30S(-S1) subunits were incubated either with 80 pmol of native S1 or with 400 pmol of FITC–S1_18_ in 50 μl TICO buffer at 37°C for 30 min. After addition of 50 μl TICO buffer the samples were applied to 100 kDa MWCO Amicon concentrators (Millipore), washed and concentrated to 50 μl by centrifugation at 10 000g. The retained fractions were subjected to SDS-PAGE analysis. After staining with SYPRO Ruby (Invitrogen) the gels were scanned employing a Typhoon using a 488 nm laser and the filters of 520 nm to detect FITC and 610 nm to detect SYPRO Ruby stained proteins, respectively.

### *In vitro* translation

The *ompA* mRNA was prepared *in vitro* as described (9). The *in vitro* translation was performed using the *E. coli* S30 Extract System for Linear Templates (Promega). The reactions containing 1 μCi/ml of [^35^S]-methionine, 0.2 μM of *ompA* mRNA and 0.3 μM of ribosomes were incubated for 60 min at 37°C in the absence or presence of 3 or 30 μM of purified proteins S1_106_, S1_87–194_ or peptide FITC–S1_18_, respectively. The reactions were stopped by addition of SDS–protein sample buffer and separated on SDS-PAGE. The dried gels were exposed to a Typhoon Molecular Dynamics PhosphoImager (GE Healthcare) for visualization and quantification.

## RESULTS

### The N-terminal S1 domain is not a *bona fide* S1 domain

We first analysed the N-terminal region of S1 (S1_106_), which is pivotal for the interaction with S2 (18), by multidimensional heteronuclear NMR. Of the 96 resonances visible on the ^1^H–^15^N HSQC spectrum (out of the 104 expected resonances) 59 could be assigned to residues Gly21 to Gly79. The remaining 37 peaks corresponding to residues Met1 to Pro20 and Phe80 to Glu106 (Figure 1B), exhibit the broadness and poor signal-to-noise ratio indicative for structural disorder in solution, which is in agreement with the results of a recent NMR study on the first domain of S1 (46). In addition, the comparison of the ^1^H–^15^N spectrum of S1_106_ with the spectra of the S1_86_ (lacking the C-terminal linker, CTL) and S1_19–86_ (lacking both, the CTL and the N-terminal segment, NTS; Figure 1B; Supplementary Figure S1A) revealed that both terminal regions are not part of the core domain, since the resonances corresponding to residues Gly21 to Gly79 remain unchanged within the three spectra (Supplementary Figure S1A). Finally, using ^1^H, ^15^N and ^13^C secondary chemical shifts we could determine that the folded core of S1_106_ comprises only four β-strands (Supplementary Figure S1B). In addition, our data show that the N- and C-terminal regions are structurally disordered when S1 is in *apo* form, i.e. when not bound to the ribosome.

### The flexible S1_NTS_ is required for ribosome binding

To dissect the role of the flexible S1_NTS_ (residues Met1 to Thr18) and the core domain of S1_106_, we analysed *in vivo* the ribosome binding capacity of the truncated protein variant (S1_19–106_; Figure 1B). Upon ectopic expression of the *rpsA*_106_ or *rpsA*_19–106_ genes in *E. coli* strain JE28, 70S ribosomes were affinity-purified as described in ‘Materials and Methods’ section. Subsequent western blot analysis revealed that in contrast to S1_106_, which completely abolishes assembly of native S1 by blocking its binding site (Figure 1C, panel a, lane 4), S1_19–106_ neither interacts with the 70S ribosome (Figure 1C, panel b, lane 2), nor interferes with binding of the native protein S1 (Figure 1C, panel a, lane 2). To further verify that the NTS is likewise vital for ribosome binding of full-length protein S1, we repeated the co-purification studies employing a full-length protein S1 lacking the NTS (S1_19–557_; Figure 1A). Here, the ectopically expressed S1 variants were detected *via* their C-terminal FLAG-tag and therefore distinguishable from the native S1. As expected, and in contrast to full length S1 (Figure 1D, panel a, lane 2), S1_19–557_ does not associate with the ribosome *in vivo* (Figure 1D, panel a, lane 4).

To further assess the role of the NTS for ribosome binding, the NTS was fused to D2–D6 of S1 (S1_NTSФ106–557_; Figure 1A). Consistently, the presence of the NTS enabled ribosome binding of protein S1_NTSФ106–557_ lacking domain D1 (Figure 1D, panel a, lane 8), whereas the flexible linker region located between domains D1 and D2 (residues 87–106) did not allow ribosome binding (Figure 1D, panel a, lane 6). Taken together, these results corroborate our assumption that the flexible NTS is the crucial element tethering S1 to the ribosome, whereas the core structure of domain D1 *per se* does not promote binding of S1 to the ribosome.

### Crystal structure of the S2–S1_NTD_ complex

Since S1 binds to the ribosome by means of protein-protein interaction *via* protein S2 (9,19), we aimed to crystallize S1_NTD_ in complex with S2. After numerous attempts, we were successful in crystallization of a chimeric protein consisting of protein S2 connected to S1_NTD_ (residues 1 to 86) *via* a five-amino acid long linker (S2–S1_NTD_; Supplementary Figure S2A). The S2–S1_NTD_ structure was solved and refined to 2–3 Å resolution (*R*_work_/*R*_free_ 18.6%/24.8%) with one molecule of S2–S1_NTD_ in the asymmetric unit. Data collection and final refinement statistics are reported in Table 1. The crystal packing analysis showed that the interaction between S2 and S1_NTD_ is formed inter-molecularly between two symmetry related molecules. The molecules are related by a crystallographic 2-fold axis, where S2 interacts with S1_NTD_ of the symmetry mate (Supplementary Figure S2B). In all subsequent structural analyses and discussions, we will refer to the S2–S1_NTD_ structure of the complex assembled from the two protomers (Figure 2A).

The S2 component retains the two domain organization consisting of a coiled-coil and an α/β globular part (Figure 2A). This structure can be superimposed with the structure of S2 in the context of the *E. coli* 30S subunit (pdb accession code: 2qbf, chain B) with a *root-mean-square deviation (rmsd)* of 1.4 Å over 212 superimposed Cα atoms. In agreement with the previous knowledge that S2 is a Zn^2+^ binding protein (47), we also identified the Zn^2+^ binding pocket within the globular domain of S2 (Figure 2A and C). The Zn^2+^ binding site in S2 is partially occupied, Zn^2+^ being octahedrally coordinated by the side-chains of residues Asp188, Asp204, Asp205 and His18 as well as two water molecules. The identity of the metal was confirmed by the presence of a characteristic peak in the anomalous difference Fourier map calculated using the data collected at wavelength 1.28 Å, corresponding to the Zn^2+^ K-edge (Supplementary Figure S2C and D).

The structure of the S1_NTD_ comprises two spatially separated structural motifs (Figure 2A): the 11 N-terminal residues of S1_NTD_ form an α-helix (from here on referred to as S1_NTH_) that is connected to the four β-stranded globular moiety (from here on referred to as S1_D1_) *via* a seven amino acid residues flexible linker. Notably, the S1_NTH_ is structurally disordered in the free form (Supplementary Figure S1) and adopts an α-helical conformation upon binding to S2 (Figure 2A and B) through a ′folding upon binding′ mechanism (48).

Interestingly, a DALI search identified the S1 domain of the RNA binding protein Tex from *Pseudomonas aeruginosa* (49) as the closest structural neighbour of S1_NTD_ (*Z*-score 5.1, rmsd of 4.9 Å over 56 equivalent Cα atoms). The S1 domain of Tex adopts the classical OB fold, and structural comparison shows that the S1_NTD_ displays a truncated OB fold missing the β-strand 5 and the N-terminal part of β-strand 1, which is in the OB fold part of both β-sheets (Supplementary Figure S2G). Further, the α-helix between β-strands 3 and 4 at the bottom of the OB fold barrel is replaced by an 11 amino acid loop. Thus, S1_D1_ is structurally distinct from other S1 domains as exemplified by the comparison with domains D4 (Supplementary Figure S2E) and D6 (Supplementary Figure S2F) of protein S1.

Surprisingly, both the S1_NTH_ and the S1_D1_ contact the globular domain of S2. The aromatic rings of two phenylalanine residues, Phe5 and Phe9 located in the S1_NTH_, form a stabilizing π-stacking interaction (50) with the aromatic ring of Phe32 located on β-strand 2 of the globular domain of S2 (Figure 2B). In addition, the core domain S1_D1_ interacts with S2 *via* two salt bridges: the side chain of Asp39 of S1_D1_ interacts with Arg208 of S2 (Figure 2C), whereas the side chain of Lys43 of S1_D1_ protrudes towards the Zn^2+^ binding pocket in protein S2 and interacts *via* polar bonds with the side chains of Asp188 and Asp205 thereby stabilizing their Zn^2+^ coordinating position (Figure 2C). Additionally, Lys43 interacts with Asn203 and the C-atom of Phe16, which is involved an aromatic stacking interaction with His15, which is in turn packing with Phe9 in S1_NTH_. To validate the likelihood of whether these interfaces mediate the interaction in solution, we performed a bioinformatics analysis with PISA (Protein Surfaces, Interfaces and Assemblies (51)). Probability measures *P*_ΔG,IF_ of specific interfaces were derived from the gain in solvation energy upon complex formation, with *P*_ΔG,IF_ > 0.5 indicating hydrophilic/unspecific and *P*_ΔG,IF_ < 0.5 to hydrophobic/specific interfaces (Supplementary Table S3). Analysis of the interface between S2 and S1_NTH_ shows that the *P*_ΔG,IF_ values (0.176, 0.311) are in the range of probabilities derived from typical protein interfaces (0.1–0.4). In the case of S2 and S1_NTD_ however, the *P*_ΔG,IF_ values >0.7 indicate a less specific interaction with a smaller interface area (Supplementary Table S3), suggesting that the dominant and specific stabilizing interaction in the complex is between S2 and S1_NTH_. Surprisingly, all protein S2 residues that are involved in interaction with S1_NTD_ are highly conserved within γ-Proteobacteria and Firmicutes (Supplementary Figure S6), despite the lack of a ribosome-bound homolog of S1 in the phylum of Firmicutes. However, this fact goes in line with the observation that *E. coli* S1 binds to *Bacillus stearothermophilus* ribosomes and greatly stimulates translation of f2 RNA (52,53).

### Cryo-EM structure of the S1_NTD_ on the ribosome

We have determined a cryo-EM structure of an *E. coli* ribosome stalled during translation of the ErmCL leader peptide (Figure 3A), at a resolution of ∼8 Å (Supplementary Figure S3A). Fitting of the crystal structure of *E. coli* 70S ribosome (40) revealed additional unassigned densities located in the cleft between the head and platform on the solvent side of the small subunit, adjacent to S2 (Figure 3A; Supplementary Figure S3B and C). We attributed these additional densities to part of S1, which was biochemically shown to be present in our ErmCL–ribosome complex (Supplementary Figure S3D). The location of the additional density is in agreement with the localization of S1 based on immunoelectron microscopy (15) and cross-linking mass spectrometry (10). Moreover, fitting the crystal structure of the chimeric S2–S1_NTD_ complex to the cryo-EM map of the ErmCL–ribosome complex based on a structural alignment between S2 from the chimeric S2–S1_NTD_ complex (yellow in Figure 3B) and S2 from the *E. coli* 70S ribosome (orange in Figure 3B) places the S1_NTD_ into one of the unassigned densities (blue in Figure 3B). Subsequently, we generated a molecular model for the complete *E. coli* S1_D1_ (Figure 3C) based on the high sequence homology with the N-terminal segment of eukaryotic initiation factor IF2α, which adopts an OB domain fold (43). After fitting of S1_D1_, an additional density remains (Figure 3C), which would be compatible in size with domain D2 of S1 (S1_D2_, Figure 3E), however, an unambiguous fitting of the OB-like fold of S1_D2_ was not possible due to the lack of resolution and apparent flexibility within this region of the map.

Notably, the additional density attributed to S1 that was recently observed in the *E. coli* SecM-stalled ribosome-channel complex (54) is in excellent agreement with our localization of S1 (Supplementary Figure S3F). In contrast, with the exception of some density for S1_NTH_, the cryo-EM map of an *E. coli* ribosome at 11.5 Å (45) reveals little or no density for S1_D1_ (Figure 3D). This was surprising since a previous localization of S1 (23) was based on a difference map between the 11.5 Å *E. coli* cryo-EM map (45) and the crystal structure of the *T. thermophilus* 30S subunit, which lacks S1 (44). In order to address this discrepancy, we re-generated a difference map between the 11.5 Å *E. coli* cryo-EM map and the crystal structure of the *T. thermophilus* 30S subunit, yielding a difference density with features similar to that reported previously (Supplementary Figure S4A). In addition, we also generated a difference map between the 11.5 Å *E. coli* cryo-EM map and the crystal structure of the *E. coli* 70S ribosome (40), which revealed that a large portion of the density attributable to S1 in the 11.5 Å *E. coli* cryo-EM map (23) was in fact due to the *E. coli* ribosomal protein S21, which is absent in the *T. thermophilus* 30S subunit (Supplementary Figure S4B). Moreover, aligning the crystal structures of the *E. coli* 70S ribosome containing mRNA and tRNAs (40) indicates that, after subtraction of density attributable to S21, the remaining density is mostly due to the mRNA and the 3′ end of the 16S rRNA (Supplementary Figure S4A and B). In contrast, when difference density maps are generated between the cryo-EM map of the ErmCL–ribosome and the *E. coli* or *T. thermophilus* 30S subunits, additional density that is not present in the 11.5 Å *E. coli* cryo-EM map, is observed that we have attributed to S1_D1_ and S1_D2_ (Supplementary Figure S4C and D). The close proximity of S1_D1_ and S1_D2_ to the 3′ end of the 16S rRNA (Supplementary Figure S4C and D) is supported by crosslinks to this region from S1 (55,56).

In addition to contacts with the mRNA and 3′ end of the 16S rRNA, the electron density of the cryo-EM map of the ErmCL-ribosome complex also reveals that S1 establishes two contacts with S2, contact one (C1) from the S1_NTH_ and an additional contact (C2) from S1_D1_ (Figure 3C). The contact C1 is consistent with the interactions between the S1_NTH_ and the β-hairpin and helix α1 of S2, and contact C2 would be compatible with the interaction observed between S1_D1_ in the vicinity of the zinc binding motif observed in the crystal structure of the chimeric S2–S1_NTD_ complex (Figure 2B and C). Thus, we believe that the interactions between S1 and S2 within the chimeric S2–S1_NTD_ complex are physiologically relevant and reflect the interactions between S1 and S2 that are observed on the ribosome.

### The π-stacking interaction between S1_NTH_ and S2 is essential for ribosome binding

**Figure 4.**
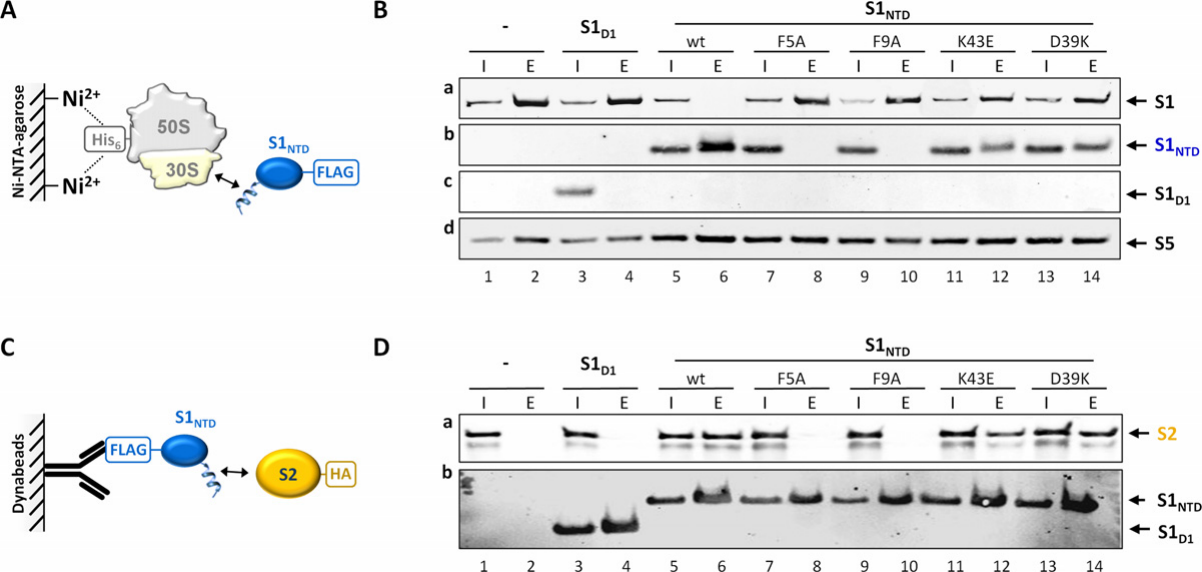
The π-stacking interaction between S1_NTH_ and S2 is pivotal for binding of S1 to the ribosome (**A** and **B**) and protein S2 (**C** and **D**). Schematic depiction of the co-purification experiments using either His-tagged ribosomes (33) (**A**) or FLAG-tagged protein S1_NTD_ variants (**C**). (**B**) Equal amounts of S30 extract (Input; lanes 1, 3, 5, 7, 9, 11 and 13) and ribosomes (Elution; lanes 2, 4, 6, 8, 10, 12 and 14) purified from *E. coli* strain JE28 before (lanes 1 and 2) and after synthesis of proteins S1_D1_ (lanes 3 and 4), S1_NTD_ (lanes 5 and 6), S1_NTD_F5A (lanes 7 and 8), S1_NTD_F9A (lanes 9 and 10), S1_NTD_K43E (lanes 11 and 12), S1_NTD_D39K (lanes 13 and 14) were tested for the presence of the respective S1 variants indicated to the right by western blot analysis using anti-S1_106_ antibodies (panels a–c). Protein S5 (panel d) served as loading control. (**D**) Under the same conditions exemplified in (B) S100 extracts were prepared and supplemented with purified HA-tagged protein S2 (input; lanes 1, 3, 5, 7, 9, 11 and 13). After incubation the FLAG-tagged protein S1 variants were immunoprecipitated by anti-FLAG antibodies (elution; lanes 2, 4, 6, 8, 10, 12 and 14; panel a) and the co-purification of protein S2 was determined by western blot analysis using anti-HA antibodies (panel b). The amounts of protein S1 variants were analysed employing anti-S1_106_ antibodies.

To determine the significance of the hydrophobic and electrostatic contacts for the S1–S2 interaction, we scrutinized the binding potential of different S1_NTD_ mutants to the ribosome (Figure 4A and B) or to purified protein S2 (Figure 4C and D). To evaluate the importance of the π-stacking interactions we removed the aromatic rings of Phe5 and Phe9 by substituting phenylalanine by alanine residues (Figure 4B and D; S1_NTD_F5A, S1_NTD_F9A). The role of the salt bridges between the core domain of S1_NTD_ and S2 to ribosome binding was assessed by charge reversal mutations of residues Lys43 and Asp39, respectively (Figure 4B and D; S1_NTD_K43E, S1_NTD_D39K). The pull down experiments using either His-tagged ribosomes (33) (Figure 4B) or FLAG-tagged S1_NTD_ variants (Figure 4D) revealed that in the absence of the π-stacking interaction *via* the aromatic rings of either Phe5 or Phe9, protein S1_NTD_ can neither interact with the ribosome (Figure 4B, panel b, lanes 8 and 10) nor with S2 (Figure 4D, panel a, lanes 8 and 10). In contrast, the mutations of residues involved in electrostatic interactions within the globular domain of S1_NTD_ exhibited only a minor effect on the assembly of S1_NTD_ to the ribosome, since the amounts of proteins S1_NTD_K43E and S1_NTD_D39K that co-purified with the ribosome (Figure 4B, panel b, lanes 12 and 14) were only slightly reduced when compared to S1_NTD_ (Figure 4B, panel b, lane 6). Correspondingly, the co-precipitation of S2 was only marginally affected when proteins S1_NTD_K43E and S1_NTD_D39K were used (Figure 4D, panel a, lanes 12 and 14). Taken together with the results shown in Figure 1, these data demonstrate that the stable S1_NTD_–S2 interaction is primarily based on π-stacking conferred by the phenylalanine residues within the S1_NTH_ and the phenylalanine residue at position 32 within the globular domain of S2. The salt bridges between S1_D1_ and the globular domain of S2 seem to play a minor role, possibly by stabilizing the interaction during a potential reorganization of the S1 structure upon mRNA binding.

### Free S1_NTS_ binds to the 30S ribosomal subunit

**Figure 5.**
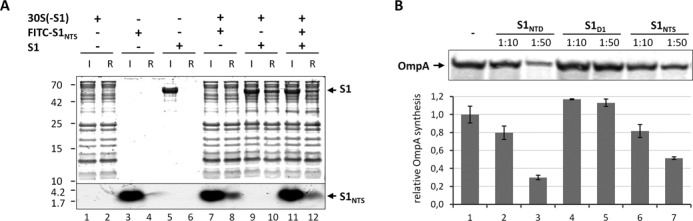
Free S1_NTS_ binds to the ribosome and interferes with translation of the canonical *ompA* mRNA. (**A**) Purified S1-depleted 30S ribosomes (30S(-S1)) were incubated in the absence (lanes 1 and 2) or in the presence of FITC labelled S1_NTS_ (lanes 7 and 8), native protein S1 (lanes 9 and 10) or both (lanes 11 and 12). Likewise, FITC labelled S1_NTS_ (lanes 3 and 4) or native S1 (lanes 5 and 6) were incubated in the absence of ribosomes. Before (input; lanes 1, 3, 5, 7, 9 and 11) and after ultrafiltration using 100 kDa MWCO Amicon concentrators (Millipore) samples were taken and the presence of the respective proteins and the S1_NTS_ peptide in the ribosome fraction (ribosome fraction; lanes 2, 4, 6, 8, 10 and 12) was determined by SDS-PAGE. (**B**) *In vitro* translation of *ompA* mRNA in the absence (lane 1) or in the presence of a 10- or 50-fold molar excess over ribosomes of S1_NTD_ (lanes 2 and 3), S1_D1_ (lanes 4 and 5) or S1_NTS_ (lanes 6 and 7), respectively. The assay was performed in triplicate and one representative autoradiograph is shown. Graph representing the quantification of three independent assays is given below. Error bars represent the standard deviation of the mean.

Given the crucial role of the S1_NTH_ in ribosome binding, we hypothesized that its interaction with S2 is the primary anchoring point for S1 on the 30S subunit of the ribosome. To corroborate this assumption we assessed whether free S1_NTS_ can interact with the 30S subunit and consequently impair binding of full length S1. To this end, we employed an ultrafiltration assay described in ‘Materials and Methods’ section using a FITC-labelled S1_NTS_ derivative to facilitate the detection of the peptide. Upon centrifugation, the 30S subunits are retained on the filter (Figure 5A, lane 2), whereas free S1_NTS_ peptide (Figure 5A, lane 4) and full-length S1 (Figure 5A, lane 6) pass through the membrane. As expected, in the presence of S1-depleted 30S subunits (30S(-S1)), the S1_NTS_ peptide (Figure 5A, lane 8) and full-length S1 (Figure 5A, lane 10) were detected in the retained ribosome fraction, indicating an interaction with the ribosomal subunit. Moreover, the concomitant addition of the FITC–S1_NTS_ peptide and protein S1 reduces the amount of both molecules in the ribosome fraction (Figure 5A, lane 12), corroborating the assumption that they compete for the same binding site on the 30S subunit.

### S1_NTS_ but not S1_D1_ inhibits translation of canonical *ompA* mRNA *in vitro*

Previously, we have shown that the synthesis of S1_NTD_ inhibits bulk mRNA translation in *E. coli in vivo* presumably because the protein binds to the ribosome and blocks assembly of native S1 (18). Given that the binding of S1 to the ribosome is dictated by the S1_NTS_, we next determined whether the S1_NTS_ peptide could also functionally interfere with canonical mRNA translation. Thus, we performed an *in vitro* translation assay employing the canonical *ompA* mRNA, translation of which is strictly dependent on the presence of S1 on the ribosome (8). As shown previously (18) and in contrast to the globular S1_D1_ lacking the S1_NTS_ (Figure 5B, lanes 4 and 5), the presence of the S1_NTD_ including S1_NTS_ interferes with translation of the *ompA* mRNA *in vitro* (Figure 5B, lanes 2 and 3). Remarkably and in line with our assumption, the addition of a 10- or 50-fold molar excess of the S1_NTS_ peptide over the ribosome likewise results in reduction of OmpA synthesis by 20 and 50%, respectively (Figure 5B, lanes 6 and 7).

## DISCUSSION

**Figure 6.**
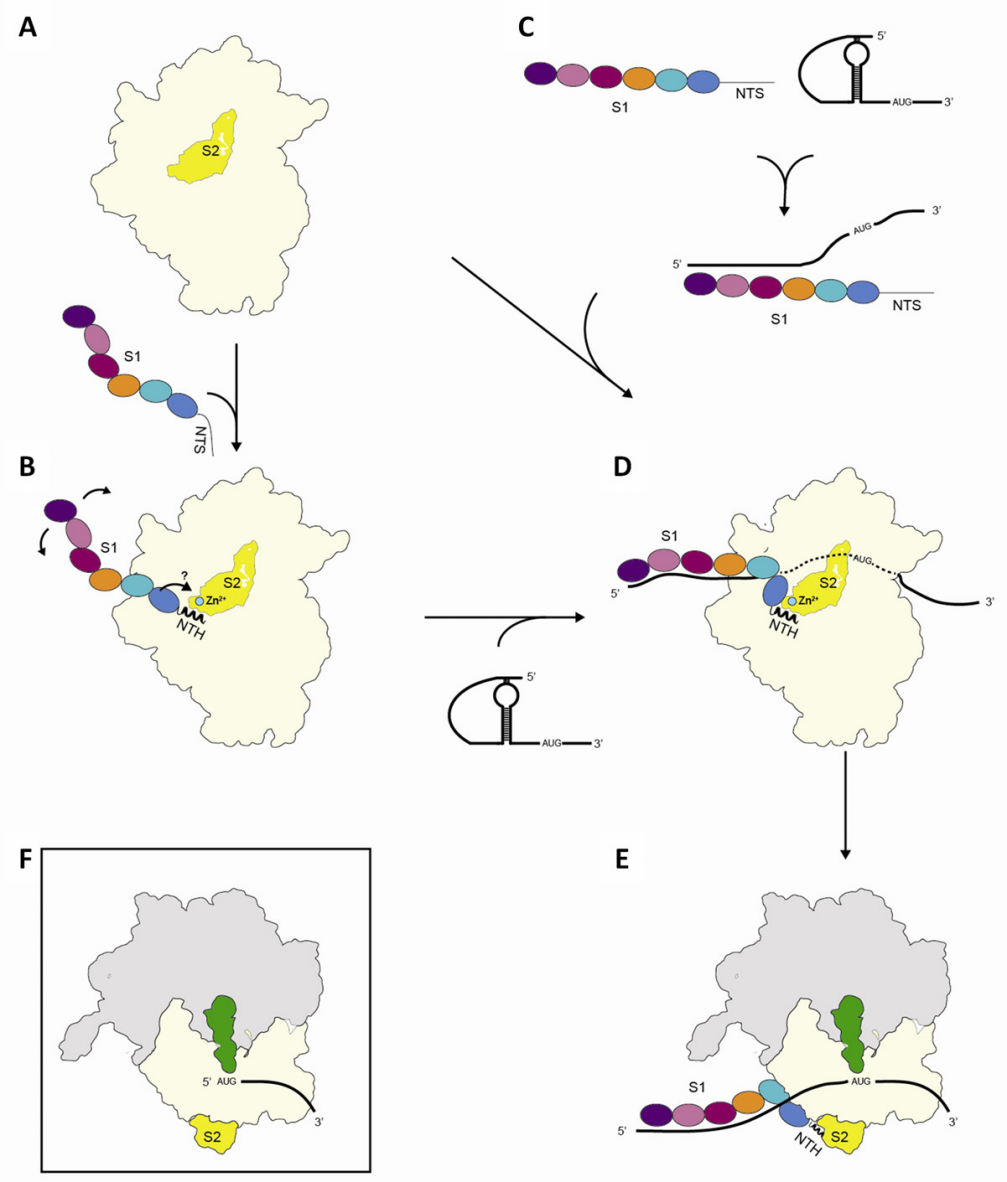
Schematic model showing the interaction of S1 with the 30S subunit. (**A**) In the free form the N-terminal segment of the multidomain protein S1 (spheres indicating the domains are colour-coded as in Figure 1A) is unstructured. (**B**) S1 can either interact with the globular domain of protein S2 (in yellow) on the 30S subunit (in light yellow) primarily *via* the N-terminal helix S1_NTH_, which adopts an α-helical conformation upon binding to S2 through a ‘folding upon binding’ mechanism (48). In this position, the protein can move in a ribosome-independent manner to scan for RNA molecules or (**C**) S1 can interact directly with the mRNA, facilitate unfolding of the mRNA, and its delivery to the ribosome (5–7). (**D**) Binding of mRNA induces a rearrangement of the S1 domains D3–D5 (12) that might facilitate the correct positioning of the mRNA possibly supported by the salt bridges between S1_D1_ and S2, leading to the formation of the (**E**) translation initiation complex. It is still in question whether the presence of the Zn^2+^ ion (green sphere) affects the affinity or the topology of S1 on the ribosome, what could potentially influence the activity or selectivity of the ribosome for specific mRNAs. (**F**) Post-translational protein modifications within the region of the S1_NTH_ or the S2 protein could likewise influence the affinity of S1 for the ribosome. Thereby, S1-depleted ribosomes that are selective for translation of lmRNAs could be present under specific conditions.

One hallmark of ribosomal protein S1 is its unique flexibility, which was suggested already more than 30 years ago (57,58). As a result, the full-length protein is not amenable to structural analysis and was thus intentionally removed from the ribosome before crystallization (44), resulting in the fact that the S1–ribosome interface hitherto remained enigmatic. Here, we present the first detailed structural analysis of the S1–S2 interface that can be rationalized in terms of the following model. The short N-terminal segment of protein S1 is intrinsically structurally disordered in solution (Figure 6A and Supplementary Figure S1A). It folds partially into a perfect helical structure upon interaction with the globular domain of S2 (Figure 6C) *via* π-stacking involving two highly conserved phenylalanine residues of S1 (Supplementary Figure S5). Due to the flexible hinge region, the protein in its elongated conformation can scan the surrounding of the ribosome for mRNA molecules, thereby increasing the sphere of ribosome action (Figure 6C). In some situations, S1 may interact with the mRNA and initiate unfolding of secondary structures in the absence of the ribosome (5,6) as well as aid in delivery of the mRNA to the ribosome (Figure 6B) (7). As revealed by NMR and SAXS analyses, binding of RNA molecules induces a topological rearrangement of the S1 domains D3–D5 (12), which might further contribute to an overall reorganization of protein S1 on the ribosome, possibly supported by the salt bridges at the boundary between the globular domains of S1 and S2, or potentially induced by the suggested interaction of the protein S1 with the 3′-terminal region of the 16S rRNA (17) (Figures 2C and 6D). Thereby, S1 could be contracted in order to position the mRNA close to the mRNA track on the 30S subunit (Figure 6D) to facilitate formation of the translation initiation complex (Figure 6E) (7).

During the preparation of this manuscript the structure of the Qβ replicase comprising the β-subunit, EF-Tu, EF-Ts and the N-terminal half of S1 was published (22). Interestingly, again domains D1 and D2 function to anchor S1 on the β-subunit and all residues involved in the interaction with the ribosome are likewise contacting the Qβ replicase. Nevertheless, several differences in the nature of interactions are evident. In contrast to the stabilizing π-stacking interaction on the ribosome, the N-terminal segment of S1 is localized in a hydrophobic pocket of the Qβ replicase, which results in an extension of the helical structure of the S1_NTH_ and concomitantly with an enlargement of the interaction surface. Likewise, the charged S1 residues, Asp39 and Lys43, that form the salt bridges with the globular domain of S2, are involved in interaction with the Qβ replicase. Asp39 interacts with two Arg residues in the replicase, whereas Lys43 is involved in the interaction with the main chain carbonyl group of Ile199. Again, this interaction surface is extended as the S1 residue Gly41 forms an additional hydrogen bond with the main chain amide group of Ile199. Taken together, these results suggest that the Qβ replicase could directly compete with S2 for the same binding sites on protein S1.

In the course of this study, we also determined the Zn^2+^ binding pocket in the globular domain of ribosomal protein S2 (Figure 2C). Among the biological relevant transition metals, zinc is peculiar as it is redox inert and shows a versatile coordination chemistry, and can hence be used as a structural or catalytic cofactor. In protein S2, the Zn^2+^ ion is coordinated in an octahedral geometry by monodentate carboxylates of three aspartic acid residues (Asp188, Asp204, Asp205), one histidine residue (His18) and two water molecules (Figure 2C). Notably, these residues are conserved across Proteobacteria and Firmicutes, indicating the importance of the presence of Zn^2+^ (Supplementary Figure S6). A search in the MESPEUS database (59) of three-dimensional metal biosites revealed the only similar coordination sphere in the l-rhamnose isomerase from *Pseudomonas stutzeri* (60), where the substrate binding site contains two metal cations. The Zn^2+^ binding site that is similar to the one found in S2 is considered to have a structural role, since it stabilizes the local structure of the protein and facilitates the correct orientation of the substrate. Thus, in the S1/S2 complex the Zn^2+^ ion might lack a catalytic activity but rather plays a structural role. However, the zinc binding pocket is located at the S1–S2 interface, with the Asp188 residue being properly positioned for the coordination of the zinc ion by the salt bridge with the Lys43 residue of S1 (Figure 2C), which resides within the highly conserved loop region connecting β-strands 2 and 3 (Supplementary Figure S5). This result raises the possibility that the S1–ribosome interaction might be modulated by the presence of Zn^2+^ ions (Figure 6C and D). Thus, besides the regulation of gene expression *via* metal responsive transcription factors, the intracellular zinc concentration could likewise affect ribosome specificity and thereby directly modulate the translatome. This hypothesis, which could add another level of complexity to the regulation of protein synthesis in response to zinc homeostasis, is currently under investigation.

Recently, evidence is accumulating that ribosome heterogeneity provides a fast and energy efficient pathway for bacteria to adapt protein synthesis to adverse conditions (18,61). In particular, several studies addressed the functional specificity of S1-depleted ribosomes for translation of lmRNAs (9,62,63). Given the formation of lmRNAs during stress conditions (61), it is conceivable that conditional post-translational protein modifications affect the small boundary between proteins S1 and S2. Thereby, the affinity of the protein for the ribosome might be reduced and S1-depleted ribosomes could be generated, which are responsible for translation of lmRNAs (Figure 6F). Concomitantly, free protein S1 might participate in other tasks, as already suggested either in the stabilization of certain transcripts (64) or in trans-translation (65). This assumption is supported by a comparative proteome analysis that revealed the differential acetylation of several r-proteins including S1 and S2 during exponential or stationary growth phase (66). Moreover, a recent study performed to decipher the phosphoproteome of *E. coli* during growth in minimal medium (67) indicates that several residues of protein S1 are differentially phosphorylated in response to the growth phase. Interestingly, the modification of residue Thr2, which is located in close proximity to the N-terminal ribosome anchoring helix, was only observed in late stationary phase. Moreover, Ser44, which is juxtapose to Lys43 that mediates the salt bridge involving the S2 zinc binding pocket, is highly phosphorylated at late stationary phase. Thus, we envisage that the negative charge introduced by the phosphorylation of Ser44 could contribute to a reorientation of Lys43, and thereby impair the formation of the respective salt bridge. This idea is supported by results indicative for the accumulation of free ribosomal proteins S1 and L7/L12 during stationary phase (68). Interestingly, the interaction of proteins L7/L12 with the ribosome is mechanistically similar to the S1-ribosome interaction. Proteins L7/L12 likewise bind to the ribosome *via* a short N-terminal domain which is connected to the functional domain by a flexible and unstructured linker (69). Notably, this N-terminal ribosome binding domain is modified in a growth phase dependent manner, which affects the stability of the interaction (70). Taken together, we hypothesize that the small boundary between proteins S1 and S2 could represent a target for modification in response to the growth-phase or environmental conditions, which might affect the affinity of S1 for the ribosome and consequently contributes to ribosome heterogeneity thereby fine-tuning protein synthesis.

## ACCESSION NUMBERS

The coordinates and the structure factors were deposited in the Protein Data Bank with accession code 4TOI and the cryo-EM map was deposited in the EMBank with accession code EMD-6211.

## SUPPLEMENTARY DATA

Supplementary Data are available at NAR Online.

SUPPLEMENTARY DATA
