# Supplementary material for: Structural basis for the interaction of protein S1 with the Escherichia coli ribosome
Source: Nucleic Acids Res. 2014 Dec 15;43(1):661–73. doi: 10.1093/nar/gku1314 (PMC4288201; doi:10.1093/nar/gku1314)
Supplement: SUPPLEMENTARY DATA [file supp_43_1_661__index.html]

Structural basis for the interaction of protein S1 with the Escherichia coli ribosome — SUPPLEMENTARY DATA 

# Structural basis for the interaction of protein S1 with the *Escherichia coli* ribosome

## SUPPLEMENTARY DATA

**Files in this Data Supplement:**

- SUPPLEMENTARY DATA
