# Supplementary material for: Structural basis for the interaction of protein S1 with the Escherichia coli ribosome
Source: Nucleic Acids Res. 2014 Dec 15;43(1):661–73. doi: 10.1093/nar/gku1314 (PMC4288201; doi:10.1093/nar/gku1314)
Supplement: SUPPLEMENTARY DATA [file supp_gku1314_nar-02548-r-2014-File008.docx]

Supplementary data

Structural basis for the flexible interaction

of protein S1 with the *Escherichia coli* ribosome

Konstantin Byrgazov^1^, Irina Grishkovskaya^2^, Stefan Arenz^3^, Nicolas Coudevylle^2^,
Hannes Temmel^1^, Daniel N. Wilson^3^, Kristina Djinovic-Carugo^2,4^ and Isabella Moll^1‡^

**Supplementary Figures**

**Supplementary Figure S1.** NMR analysis of proteins S1_106_, S1_86_, and S1_19-86_. (**A**) Left panels: comparison of the 1H-15N HSQC spectra of proteins S1_106_ (red) and S1_86_ (blue). Right panels: overlay of the spectra of proteins S1_86_ (blue) and S1_19-86_ (green). The bottom panels show close up views of the respective areas indicated by the dashed boxes. (**B**) Protein sequence alignment of S1_106_ and S1 domain D4. The position of the four β-strands of protein S1_106_ identified by NMR analysis are indicated by blue arrows. The red arrows indicate the positions of the β-strands of domain D4.

**Supplementary Figure S2.** Crystal structure analysis of the chimeric S2-S1_NTD_ protein. (**A**) Amino acid sequence of the chimeric S2-S1_NTD_ protein used for the crystal structure analysis. The sequences corresponding to protein S2 (yellow), the flexible linker (black and italics) and protein S1_NTD_ (blue) are indicated. (**B**) Dimer of the chimeric S2-S1_NTD_ protein formed by inter-molecular interaction, where S2 interacts with S1_NTD_ of the symmetry mate. S2 and S1_NTD_ of one monomer are indicated in yellow and blue, and of the second monomer in light and dark grey, respectively. (**C**) Anomalous difference Fourier map showing the position of the Zn^2+^ ion contoured at 3.5 σ. The anomalous data was collected to 3 Å resolution at ID23-1 ESRF (Grenoble, France) at wavelength 1.28 Å (9.68 keV). (**D**) X-ray energy scan around the absorption edge of Zn (red) and its first derivative depicted in blue. The energy is given in keV. Comparison of the structure of protein S1_NTD_ with the domains D4 (**E**) and D6 (**F**) of protein S1 from *E. coli* and the S1 domain of the RNA binding protein Tex from *P. aeruginosa* (**G**).


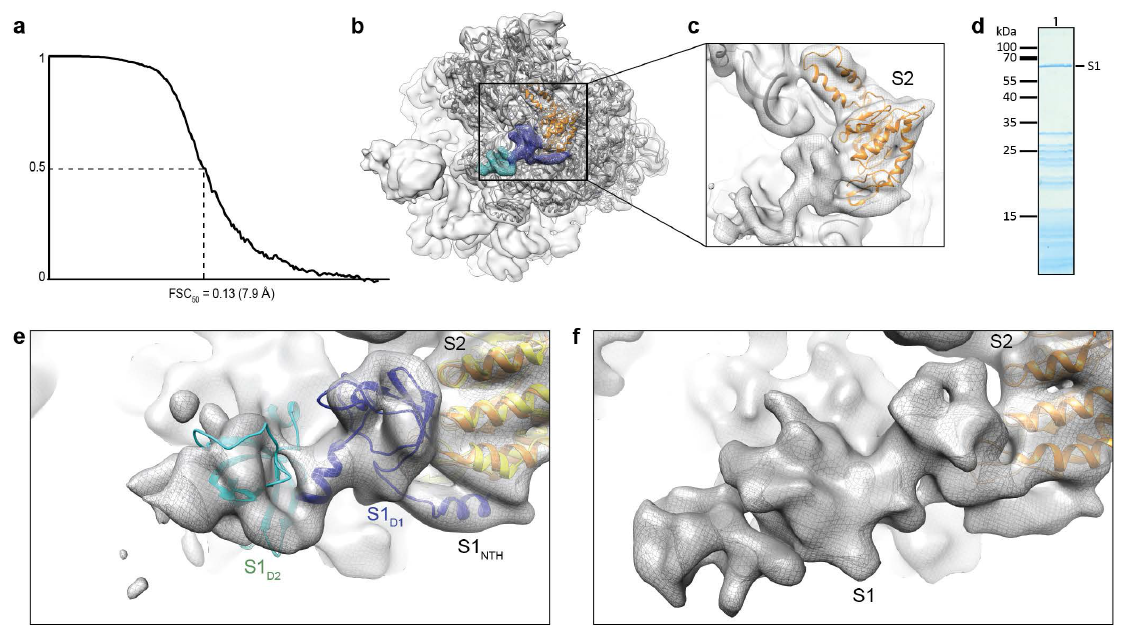


**Supplementary Figure S3.** Binding position of S1 on the *E. coli* 70S ribosome. (**A**) The average resolution of the cryo-EM map of the ErmCL-ribosome complex was 7.9 Å as determined using the Fourier shell correlation (FSC) cut-off value of 0.5. (**B**) Overview of the cryo-EM map of the ErmCL-ribosome complex (grey mesh), with rigid-body fitted crystal structure of the 30S subunit (pdb accession code 3ofo) from the *E. coli* 70S ribosome (1) (dark grey). Ribosomal protein S2 is colored orange and highlighted electron densities for S1_D1_ (blue) and S1_D2_ (cyan). (**C**) Inset from (B) showing extra unaccounted for density in the cryo-EM map of the ErmCL-ribosome complex (grey mesh) adjacent to S2 (orange). (**D**) InstantBlue (Expedeon) stained 15% SDS polyacrylamide gel electrophoresis of the ErmCL-ribosome complex, indicating the presence of ribosomal protein S1 at ~60 kDa. (**E**) Cryo-EM map (grey mesh) of the ErmCL-ribosome complex containing additional density for domain 1 (S1_D1_, blue) and domain 2 (S1_D2_, cyan) of ribosomal protein S1. The model for S1_D1_ was obtained by aligning S2 (yellow) of the chimeric S2-S1_NTD_ with S2 (orange) from an *E. coli* 30S subunit (pdb accession code 3ofo) (1) fitted to the cryo-EM map (grey mesh) as a rigid body. The model was refined for the complete S1_NTD_ based on homology with eIF2α (pdb accession code 1kl9) (2) and fitted so as to maintain interactions between S1 and S2 as observed in the chimeric crystal structure, but also constrained by the electron density of the cryo-EM map (grey mesh). A tentative model for S1_D2_ (cyan) was generated based on homology with eIF2α (pdb accession code 1kl9) (2) and fitted into the density. (**F**) Electron density map (grey mesh) of a SecM-stalled ribosome-channel complex (3) with fitted crystal structure for S2 (orange) from an *E. coli* 30S subunit (pdb accession code 3ofo) (1).


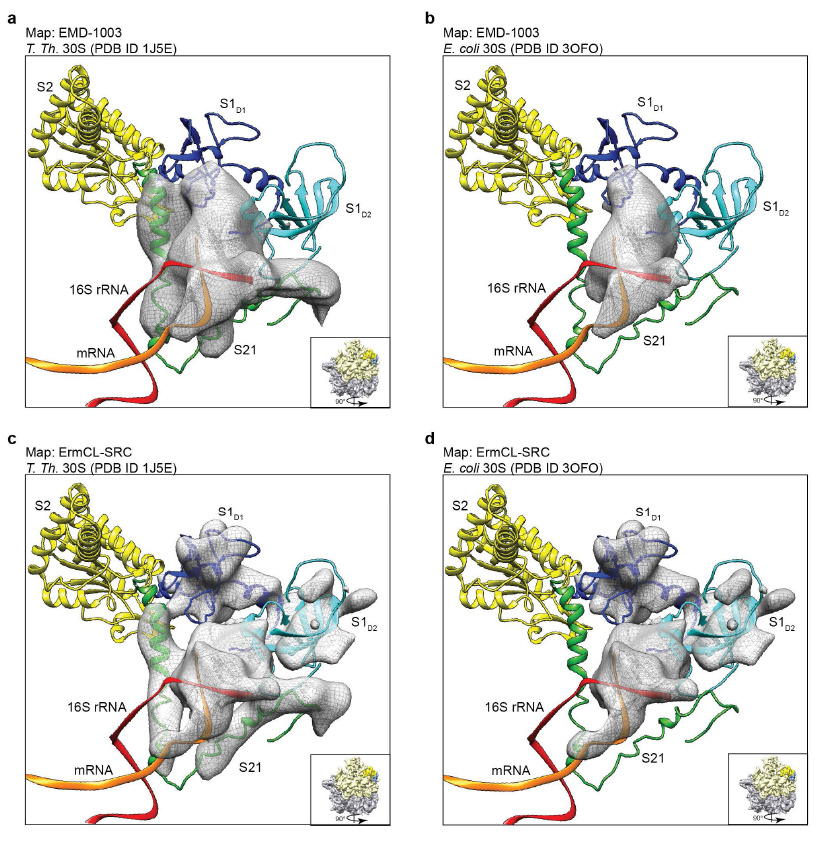


**Supplementary Figure S4.** Comparison of cryo-EM electron densities for S1 on the *E. coli* 70S ribosome. Difference electron density maps (grey mesh) calculated by **(A)** subtracting the filtered map for *T. thermophilus* 30S subunit (PDB ID 1J5E (4)) from EMD-1003 (5), or from **(B)** the ErmCL-SRC map as well as by subtracting the filtered map for *E. coli* 30S subunit (pdb accession code 3ofo (1)) from **(C)** EMD-1003 (5), or from **(D)** the ErmCL-SRC map. The relative positions of ribosomal protein S21 (green) (pdb accession code 3ofo (1)), 16S rRNA (red) and mRNA (orange) from (pdb accession code 4gd2 (6)), as well as S2 (S1_NTD_-S2; yellow), S1 domain 1 (S1_NTD_-S2; blue) and domain 2 (tentative placement of model, which was generated based on homology with eIF2α (pdb accession code 1kl9 (2), cyan).


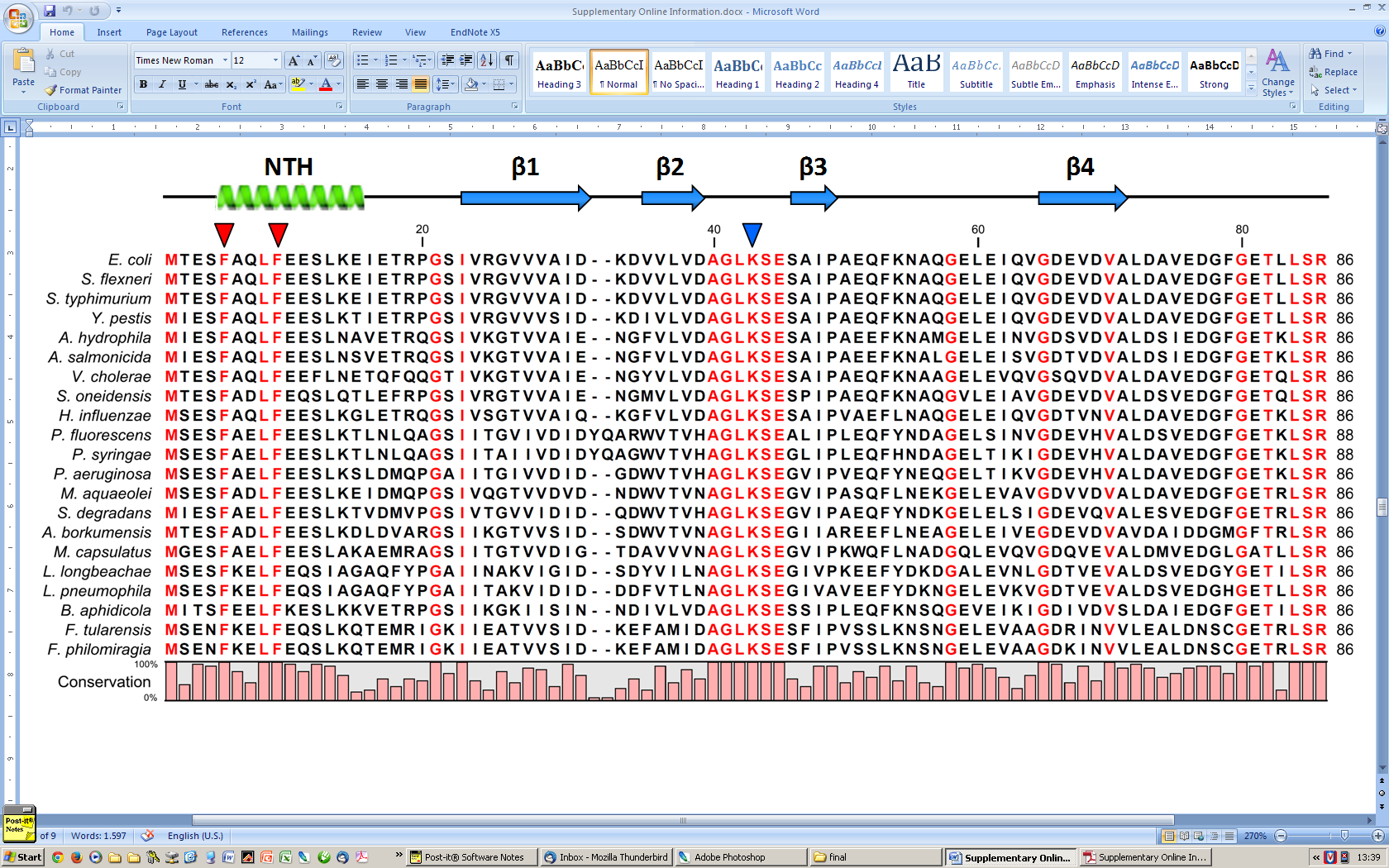


**Supplementary Figure S5.** Multiple sequence alignment of the S1_NTD_ protein of several representatives of the class of γ-Proteobacteria. The 100% conserved residues are indicated in red. The Phe5 and Phe9 residues involved in the π-stacking interaction with S2 and the Lys43 residue contacting the zinc binding pocket of S2 are marked by red and blue arrow heads, respectively. The position of the NTH (green helix) and the four β-strands (blue arrows) as determined by crystallography are indicated above. The amino acid alignment was generated using CLC Genomics Workbench software [http://www.clcbio.com/].

**Supplementary Figure S6.** Multiple sequence alignment of the S2 protein of several representatives of the γ-Proteobacteria and Firmicutes. The 100% conserved residues are indicated in red. The residues involved in zinc-binding are indicated by red arrows and the residues contacting the S1_NTD_ are indicated by green arrows. The amino acid alignment was generated using CLC Genomics Workbench software [http://www.clcbio.com/].

**Supplementary Tables**

**Supplementary Table S1.** Bacterial strains and plasmids used in this study

___________________________________________________________________________­­__

Relevant features Source or reference

***E. coli* strains:**

JE28 MG1655::*rplL-his* (7)

Tuner F^–^ *ompT hsdS*_B_ (r_B_^–^ m_B_^–^) *gal dcm lacY1* Novagen

Tuner(DE3) F^–^ *ompT hsdS*_B_ (r_B_^–^ m_B_^–^) *gal dcm lacY1(DE3)* Novagen

**Plasmids:**

pProEX-HTb vector for Trc driven gene expression Invitrogen

pProEX-S2-HA encodes his- and HA-tagged S2 WT this study

pProEX-S2-S1_NTD_ encodes his-tagged S2-S1_NTD_ this study

pPro-S1_86_F encodes FLAG-tagged S1_86_ this study

pPro-S1_106_F encodes FLAG-tagged S1_106_ (8)

pPro-S1_19-86_F encodes FLAG-tagged S1_19-86_ this study

pPro-S1_19-106_F encodes FLAG-tagged S1_19-106_ this study

pPro-S1F encodes FLAG-tagged S1 (8)

pPro-S1_19-557_F encodes FLAG-tagged S1_19-557_ this study

pPro-S1_87-557_F encodes FLAG-tagged S1_87-557_ (8)

pPro-S1_NTSΦ106-557_ encodes FLAG-tagged S1_NTSΦ106-557_ this study

pPro-S1_86_F_F5A_ encodes FLAG-tagged S1_86_, F5A this study

pPro-S1_86_F_F9A_ encodes FLAG-tagged S1_86_, F9A this study

pPro-S1_86_F_D39K_ encodes FLAG-tagged S1_86_, D39K this study

pPro-S1_86_F_K43E_ encodes FLAG-tagged S1_86_, K43E this study

pET22b vector for T7 driven over expression Novagen

pET-S1_106_  pET derivative encoding for his-tagged S1_106_ this study

pET-S1_86_ pET derivative encoding for his-tagged S1_86_ this study

pET-S1_19-86_ pET derivative encoding for his-tagged S1_19-86_ this study

pKS0325 *ompA* gene under control of T7-promoter (9)

____________________________________________________________________________

**Supplementary Table S2.** Oligonucleotides used in this study

| **P1** | TATAGGCGCCGAATTCGATGCAACTGTTTCC | fwd primer to clone S2-HA |
| --- | --- | --- |
| **P2** | TATACTCGAGTTAAGCGTAATCTGGAACATCGTACTCAGCTTCTACG | rev primer to clone S2-HA |
| **P3** | TATAAAGCTTATATTTTCAGGGTGAATCTTTTGCTCAACTC | fwd primer to clone S2-S1NTD |
| **P4** | TATAAAGCTTTTACAGCAGAGTTTCACCG | rev primer to clone S2-S1NTD |
| **P5** | P-GAAACTGTTACCGGTGTTATC | fwd primer to remove S1_D1_-coding sequence |
| **P6** | P-ACCCGGGCGGGTTTCG | rev primer to remove S1_D1_-coding sequence |
| **P7** | P-CCGGGTTCTATCGTTCG | fwd primer to remove the first 18 codons of *rpsA* |
| **P8** | P-CATGGTCTGTTTCCTGTG | rev primer to remove the first 18 codons of *rpsA* |
| **P9** | P-GACTATAAGGATGACG | fwd primer to remove the sequence coding for S1_87-106_ |
| **P10** | P-CAGCAGAGTTTCAC | rev primer to remove the sequence coding for S1_87-106_ |
| **P11** | TATACATATGACTGAATCTTTTGCTC | fwd primer to amplify *rpsA* from the 1^st^ codon |
| **P12** | TATACATATGACCCGCCCGGGTTC | fwd primer to amplify *rpsA* sequence from the 19th codon |
| **P13** | TATACTCGAGTTCAGCATCTTCGTAAGC | rev primer to amplify *rpsA* until 106^th^ codon |
| **P14** | TATACTCGAGCAGCAGAGTTTCAC | rev primer to amplify *rpsA* until 86^th^ codon |
| **P15** | P-CTCTTCAAAGAGTTGAGCGGCAGATTCAG | rev primer to introduce the mutation F5A |
| **P16** | P-CTCTTCCGCGAGTTGAGCAAAAGATTCAG | rev primer to introduce the mutation F9A |
| **P17** | P-TCCTTAAAAGAAATCGAAACCCGCCCG | fwd primer to introduce the mutations F5A and F9A |
| **P18** | P-GTTAAAGCTGGTCTGAAATCTG | fwd primer to introduce the mutation D39K |
| **P19** | P-GTTGACGCTGGTCTGGAATCTG | fwd primer to introduce the mutation K43E |
| **P20** | P-CAGTACTACGTCTTTGTCGATAG | rev primer to introduce the mutations D39K and K43E |

**Supplementary Table S3.** The PISA interface analysis.

|  | **S2** | **S1_NTD_** | **S2/S1_NTH_** | **S1_NTH_** | **S2/S1_D1_** | **S1_D1_** |
| --- | --- | --- | --- | --- | --- | --- |
| **Number of atoms** | 90 (4.8)% | 81 (16.8%) | 48 (2.6%) | 42 (13.6%) | 42 (2.2%) | 39 (11.1%) |
| **Number of residues** | 22 (9.2%) | 23 (35.9%) | 12  (5%) | 12  75%) | 11  (4.6%) | 11 (22.9%) |
| **Solvent-accessible area (Å^2^)** | 725 (5.6%) | 870 (15.7%) | 402.3 (3.1%) | 456.6 (25.7%) | 322.7 (2.5%) | 413.5 (11%) |
| **Solvatation energy gain [kcal/mol]** | -2  (0.9%) | -4.8 (10.9%) | -2.3 (1.1%) | -5.5 (83.2%) | 0.3 (0.1%) | 0.7  (-1.9%) |
| **P-value** | 0.495 | 0.397 | 0.311 | 0.176 | 0.703 | 0.818 |

**Supplementary References**

1. Dunkle, J.A. and Cate, J.H. (2010) Ribosome structure and dynamics during translocation and termination. *Annu. Rev. Biophys.*, **39**, 227-244.

2. Nonato, M.C., Widom, J. and Clardy, J. (2002) Crystal structure of the N-terminal segment of human eukaryotic translation initiation factor 2alpha. *J. Biol. Chem.*, **277**, 17057-17061.

3. Park, E., Menetret, J.F., Gumbart, J.C., Ludtke, S.J., Li, W., Whynot, A., Rapoport, T.A. and Akey, C.W. (2014) Structure of the SecY channel during initiation of protein translocation. *Nature*, **506**, 102-106.

4. Wimberly, B.T., Brodersen, D.E., Clemons, W.M., Jr., Morgan-Warren, R.J., Carter, A.P., Vonrhein, C., Hartsch, T. and Ramakrishnan, V. (2000) Structure of the 30S ribosomal subunit. *Nature*, **407**, 327-339.

5. Gabashvili, I.S., Agrawal, R.K., Spahn, C.M., Grassucci, R.A., Svergun, D.I., Frank, J. and Penczek, P. (2000) Solution structure of the *E. coli* 70S ribosome at 11.5 A resolution. *Cell*, **100**, 537-549.

6. Dunkle, J.A., Wang, L., Feldman, M.B., Pulk, A., Chen, V.B., Kapral, G.J., Noeske, J., Richardson, J.S., Blanchard, S.C. and Cate, J.H. (2011) Structures of the bacterial ribosome in classical and hybrid states of tRNA binding. *Science*, **332**, 981-984.

7. Ederth, J., Mandava, C.S., Dasgupta, S. and Sanyal, S. (2009) A single-step method for purification of active His-tagged ribosomes from a genetically engineered *Escherichia coli*. *Nucleic Acids Res.*, **37**, e15.

8. Byrgazov, K., Manoharadas, S., Kaberdina, A.C., Vesper, O. and Moll, I. (2012) Direct interaction of the N-terminal domain of ribosomal protein S1 with protein S2 in *Escherichia coli*. *PloS One*, **7**, e32702.

9. Ried, G., Koebnik, R., Hindennach, I., Mutschler, B. and Henning, U. (1994) Membrane topology and assembly of the outer membrane protein OmpA of *Escherichia coli* K12. *Mol. Gen. Genet.*, **243**, 127-135.
